# A comprehensive analysis of factors influencing CO_2_-oil displacement in sandstones under reservoir conditions by online nuclear magnetic resonance testing

**DOI:** 10.1038/s41598-026-54683-x

**Published:** 2026-05-24

**Authors:** Dachao Qi, Tianran Ma, Cai Li, Chaobin Guo, Yong Yuan, Zeng Wang

**Affiliations:** 1 Qingdao Institute of Marine Geology, China Geological Survey, Qingdao, 266237 Shandong China; 2 Laboratory for Marine Mineral Resources, Qingdao Marine Science and Technology Center, Qingdao, 266237 Shandong China; 3 State Key Laboratory Geomechanics and Deep Underground Engineering, University of Mining and Technology, Xuzhou, 221116 Jiangsu China; 4 School of Mechanics and Civil Engineering, China University of Mining and Technology, Xuzhou, 221116 China; 5 Chinese Academy of Geological Sciences, Beijing, 100094 China

**Keywords:** CO_2_-oil displacement, Sandstone, Reservoir conditions, Online NMR testing, Pore structure, Energy science and technology, Engineering, Solid Earth sciences

## Abstract

Carbon Capture, Utilization and Storage-Enhanced Oil Recovery (CCUS-EOR) technology can effectively improve crude oil recovery while simultaneously sequestering CO_2_ in geological formations to reduce emissions. The distribution and migration patterns of oil and gas in reservoir rock masses remain one of the key issues worthy of study, among which CO_2_-oil displacement experiments are the most direct research method. Nuclear Magnetic Resonance (NMR) provides technical assurance for the visualization and quantification of displacement experiments. Different from previous studies, we selected experimental cores based on their pore characteristics and optimized the experimental scheme. Strictly controlled single variables were used to determine the effects of various factors, including injection pressure, temperature, pore structure, permeability, and crude oil viscosity, on CO₂ flooding efficiency. An in-depth analysis of the oil and gas migration mechanisms during CO_2_ flooding was conducted using T_2_ relaxation spectra and oil-filled porosity distribution maps. The study identified oil recovery has nonlinear relationship with injection pressure and near-linear relationships with temperature, compared the displacement differences between medium and high viscosity oils, and revealed the dominant role of pore structure on oil recovery effectiveness. It is worth noting that the order-of-magnitude change in permeability did not significantly improve oil recovery, which is quite different from previous studies.

## Introduction

The massive emissions of greenhouse gases like CO_2_ have led to global warming, significantly impacting the human living environment in recent years^1,2^. Studies have shown that CCUS (Carbon Capture, Utilization, and Storage) technology, which captures and sequesters CO_2_ in geological formations to reduce emissions, can yield economic benefits while achieving social benefits^3–5^. CO_2_ can exist in a supercritical state under the formation conditions of most oil reservoirs, largely dissolves into crude oil to form a miscible fluid, causing oil swelling, effectively reducing oil viscosity to promote flow, and improving reservoir properties to enhance oil recovery^6,7^. Using CO_2_ as a displacement medium for oil extraction is feasible and effective, and CCUS-EOR projects have become the most widely applied method for CO_2_ geological storage worldwide^8,9^.

Laboratory experiment is the most direct and intuitive method to study the phase interaction between CO_2_ and crude oil in the reservoir and reveal the migration and diffusion law of two-phase fluid^10–12^. Conventional displacement experiments can obtain oil recovery efficiency under different conditions, but cannot describe the internal oil and gas migration patterns within the core. NMR testing, as a non-destructive monitoring technique, allows for real-time monitoring and analysis of the internal micro-pore structure of the core and the distribution and migration of various fluid phases, offers a new method for visualizing the displacement process^13,14^. Combining NMR monitoring and VOF simulation methods, exploring the multiphase flow mechanisms of CO_2_ in low-permeability reservoir rocks, finding that CO_2_ miscible displacement can improve oil recovery efficiency and CO_2_ storage capacity^15^. Wang et al.^16^, through core flooding and NMR imaging experiments, comprehensively studied the key parameters of CO_2_ miscible flooding, the influence of CO_2_ miscible flooding additives, and the dominant enhanced oil recovery mechanisms under different pressures. Baban et al.^17^, using NMR technology, studied CO_2_ measurement, permeability profiles, pore size distribution, and the effects of CO_2_ residual trapping in oil-wet sandstones, providing important fundamental theory and experimental data for CO_2_ trapping and storage in oil-wet sandstones. Dynamically monitoring multiphase flow and pore-fracture behavior during CO_2_/water flooding in ultra-low permeability reservoirs by online LF-NMR technology, found that in CO_2_ flooding, oil recovery is primarily contributed by percolation pores and migration pores, whereas in water flooding, all pores contribute almost equally to oil recovery^18^. Research on the effects of in-situ stress and displacement pressure on CO_2_ and oil flow distribution with the analysis by online NMR system, indicated that increased in-situ stress significantly reduces oil mobility and recovery in ultra-low permeability reservoirs, and provided important insights for enhanced oil recovery^19^. Xing et al.^20^ selected three typical core samples with different mineral types for CO_2_ flooding experiments and used NMR testing technology to investigate the potential relationship between mineral content and tight oil recovery, revealing fundamental understanding of the enhanced oil recovery mechanisms of CO_2_ flooding related to mineral composition. Low-flied NMR also had successful application in quantifying capillary trapping by using transverse relaxation time (T_2_) measurements to determine the content of residual water and the surface-to-volume ratio distribution of the pore space occupied by the residual water^21^. Correlating changes in T_2_ time distributions during core imbibition with the displacement of saline solution in pores of different sizes, proving that NMR T_2_ measurements can provide an average probability distribution of pore size and the proportion occupied by the water phase in the core^22^.

In CO_2_ flooding experiments, pressure and temperature are external factors affecting oil and gas migration and recovery, while the internal effect factors are mainly related to the pore structure and permeability of cores^23–25^. Furthermore, the mechanisms of oil-gas interaction vary under different displacement methods^26,27^. Studying the effects of different injection pressures and cycle numbers on remaining oil distribution and production during CO_2_ gas injection and production processes by NMR technology, determining the distribution of remaining oil across different pore size ranges, indicating that higher injection pressures positively affect oil recovery from small pores and blind pores^28^. Increasing matrix permeability can improve oil recovery, reduce the adverse effects of fractures, and provide a useful reference for the development of tight reservoirs^29^. Using low-field NMR tests to monitor oil distribution during CO_2_ flooding and CO_2_ oil-based foam flooding, finding that fracture aperture and matrix permeability affect the oil production capacity of oil-based foam flooding, and there are also differences in the oil enhancement effects between CO_2_ flooding and oil-based foam flooding for non-fractured cores^30^.

Although researchers have conducted extensive and beneficial explorations in the field of CO_2_ flooding experiments, some current experimental schemes are not comprehensive enough and still have design flaws, making it difficult to characterize the influence of a single factor on the experimental results well, especially concerning factors such as core permeability and pore structure^31–33^. Therefore, based on the pore characteristics of reservoir cores, this study further optimized the experimental scheme, strictly controlled single variables, and comprehensively and accurately investigated the effects of a series of factors, including injection pressure, temperature, pore structure, permeability, and oil viscosity, on CO_2_ flooding. Based on the comparison of experimental results, it was determined that pore structure plays a dominant role among various factors, while the magnitude change in permeability within the same pore structure type does not significantly enhance oil recovery, a phenomenon distinctly different from previous research findings.

## Experimental principle

The Nuclear Magnetic Resonance testing for CO_2_ flooding can generally be divided into two modes: offline testing and online testing. Offline testing involves removing the core from the holder after a period of gas flooding or a certain volume of gas injection for NMR testing, which can effectively avoid most noise interference on the test results, while online testing involves directly performing NMR tests on the core within the holder throughout the entire displacement process to obtain relaxation information and NMR imaging, allows NMR scanning of the core while maintaining certain pressure and temperature conditions, avoiding the impact of temperature and pressure changes on fluid distribution within the core.

**Fig. 1:**
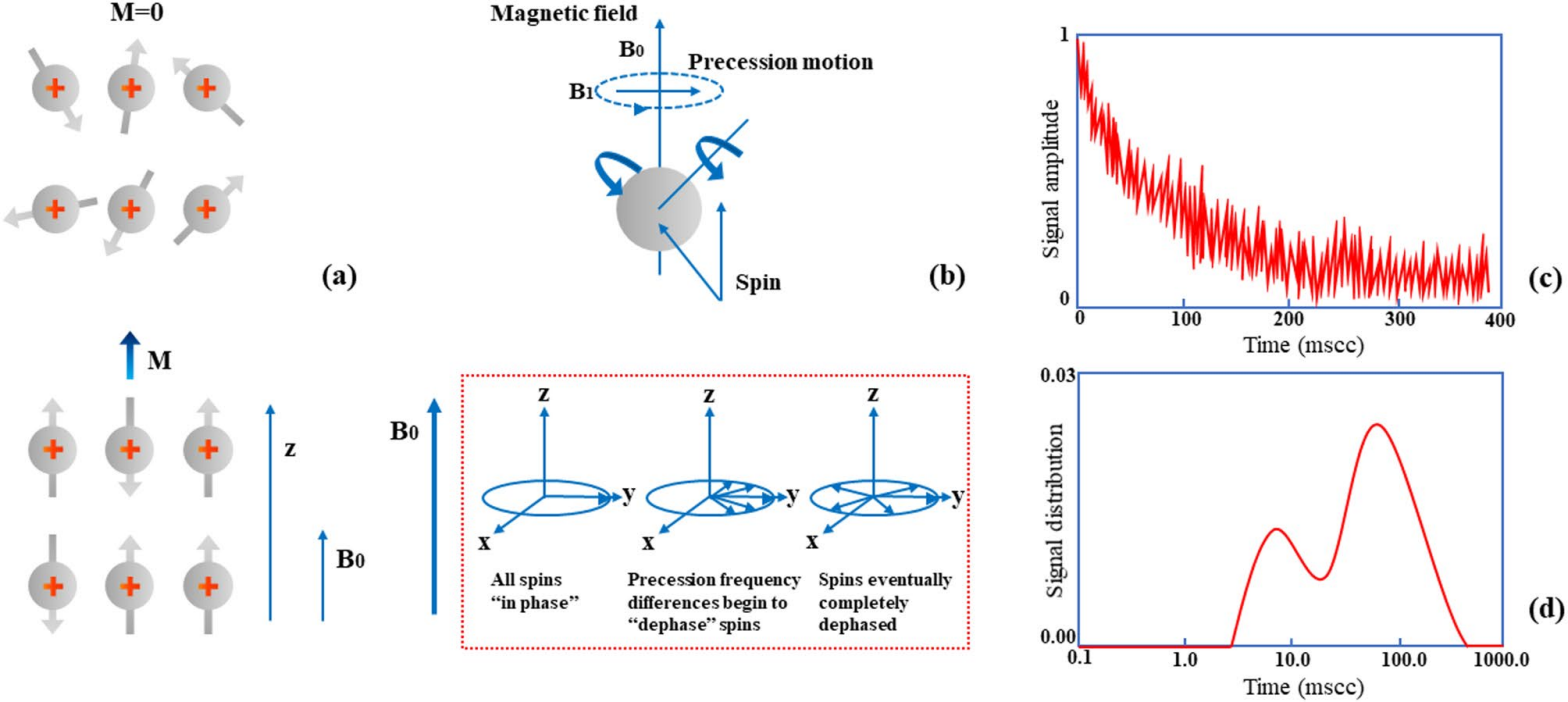
Nuclear magnetic testing process: (**a**) Proton arrangement; (**b**) Proton rotation; (**c**) Raw data collection; (**d**) Data post-processing.

The essence of NMR testing technology is the acquisition and processing of proton information. As shown in Fig. 1, the protons in the fluids within the core are randomly oriented before NMR detection, when detection begins, the testing equipment first emits a constant magnetic field, causing the protons to develop a magnetization vector along the direction of the magnetic field. Then, an alternating electromagnetic field is generated to cause the protons to process, and return to their equilibrium positions when all magnetic fields are removed, an entire process is called relaxation. During relaxation, the CPMG sequence generates a series of spin echoes, which form the raw NMR data (Fig. 1(c)) and from which the final T_2_ (transverse relaxation time) distribution can be obtained through fitting.

For fluids in pores, *T*_*2*_ consists of three parts: the diffusion relaxation time *T*_*2k*_, the free relaxation time *T*_*2Z*_ and the surface relaxation time *T*_*2B*_^34^:

1 $$\:\frac{1}{{T}_{2}}=\frac{1}{{T}_{2\mathrm{k}}}+\frac{1}{{T}_{2\mathrm{Z}}}+\frac{1}{{T}_{2\mathrm{B}}}$$

When the pore contains only one type of wetting fluid, the diffusion relaxation time and free relaxation time can be neglected:

2 $$\:\frac{1}{{T}_{2}}\approx\:\frac{1}{{T}_{2\mathrm{B}}}={\rho\:}_{2}\left(\frac{S}{V}\right)$$

Where $\:{\rho\:}_{2}$ is the surface relaxivity, *\:S/V* is the surface-to-volume ratio of the pore.

### Experimental materials and apparatus

#### Experimental materials

**Fig. 2:**
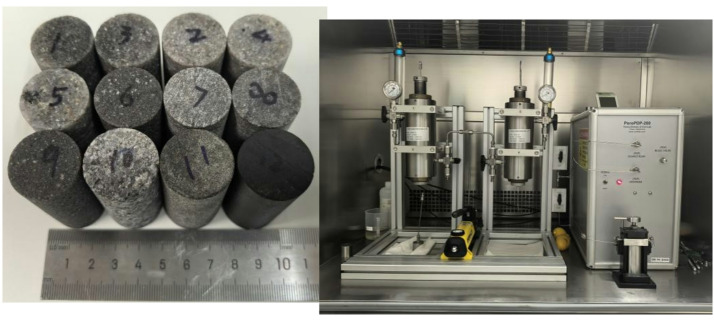
Sandstone samples (1 ~ 12) and testing equipment used for permeability testing.

The sandstones used in this study were sourced from Shanxi Province, China, with a burial depth range of 1280 ~ 1420 m. According to the standard for test methods of engineering rock masses, the drilled sandstone samples were processed into cylindrical specimens of Φ25mm × 50 mm, as shown in Fig. 2, were labeled 1, 2, 3, … 12, and gas permeability testing was performed on the cores using a pulse decay permeameter (PoroPDP-200).

The test results are shown in Table 1. Except for core 12, which had a gas-measured porosity of only 0.23%, the gas-measured porosity of the other core samples ranged from 3.14% to 10.46%, indicating significant heterogeneity in the rock formation across the reservoir area. Analyzing the test results, cores 1, 2, and 3 had relatively high gas-measured porosity and permeability, all above 3.5 md, suggesting good connectivity between pores in these three cores, and the pore types should be dominated by medium to large pores, the gas permeability of cores 4 ~ 12 was all below 1 md in contrast. Except for core 12, which had a very dense structure with minimal pore space, the remaining core samples mostly exhibited high gas-measured porosity but low gas permeability, which indicates that although these cores have relatively large pore space, the internal pores are predominantly small and micropores, and the connectivity between pores is poor.

**Table 1:** Properties of sandstone samples used in the experiment.

Core No.	Length (mm)	Diameter (mm)	Hydrocarbon porosity (%)	Gas porosity (%)	Gas permeability (md)	Used for experiment
1	50.37	24.86	-	10.46	4.64	-
2	50.42	25.02	8.86	9.37	3.90	√
3	50.26	25.02	-	10.43	3.54	-
4	50.19	25.02	5.79	5.63	0.03	√
5	50.27	24.97	-	6.30	0.15	-
6	50.34	24.56	7.02	7.46	0.25	-
7	50.29	24.51	3.00	3.14	0.02	√
8	50.67	24.54	4.00	4.42	0.02	√
9	50.24	24.5	6.44	6.64	0.27	√
10	50.18	25.27	6.13	6.27	0.03	√
11	50.09	25.30	-	4.36	0.10	-
12	50.20	25.22	-	0.23	0.07	-

According to the experimental plan, cores 2, 4, 7, 8, 9, and 10 were selected for the CO_2_ flooding NMR tests. For the injection pressure group, three cyclic experiments were conducted using Core 10, with the experiments designated as 10 − 1, 10 − 2, and 10 − 3, respectively. Core 9 was used for the temperature group following the same experimental procedure. Since Cores 9 and 10 have comparable permeability but different pore structures, they were also selected as the experimental cores for the pore structure group. Cores 2, 4, and 9 exhibit similar pore structures but differ in permeability by orders of magnitude, thus, these three cores were chosen for the permeability group to effectively minimize the influence of pore structure. Cores 7 and 8 have similar pore structures and permeability, and were therefore used as the oil viscosity group, saturated with oils of different viscosities. The oil samples used in the experiments were #46 white oil (medium viscosity oil, viscosity approximately 50mPa·s at 40 °C) and #100 white oil (high viscosity oil, viscosity approximately 100mPa·s at 40 °C), with a density of 0.8 ~ 0.9 g/cm^3^, and the gas used in this experiment was 99% pure CO_2_.

#### Experimental apparatus

**Fig. 3:**
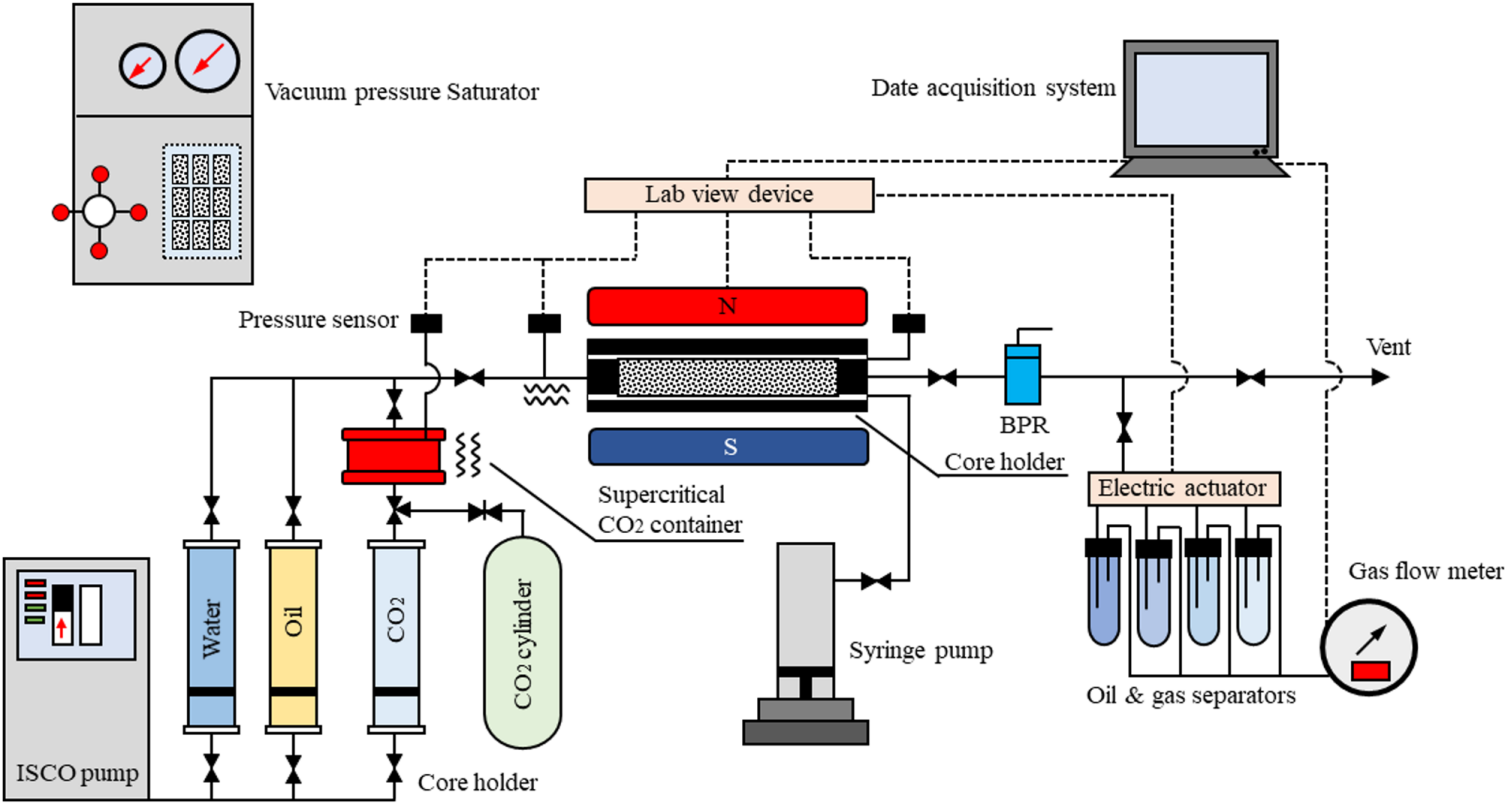
Schematic diagram of carbon dioxide oil displacement nuclear magnetic resonance experimental device.

The low field nuclear magnetic resonance (NMR) device for CO_2_ flooding was used for oil displacement test to study the change of wellhead pressure and the distribution of remaining oil during oil displacement, and to reveal the law of CO_2_ flooding in sandstone cores and the effect of different experimental conditions on recovery. Figure 3 shows the schematic diagram of NMR displacement device, mainly consists of a vacuum pressure saturation system, injection system, core holder system, temperature control system, nuclear magnetic resonance testing system, metering system, and data acquisition and analysis system. The vacuum pressure saturation system is mainly used for core saturation, with a maximum saturation pressure of 60 MPa; the injection system includes an ISCO injection pump (maximum pressure 10000 psi, accuracy 0.2%, minimum flow rate 0.78 × 10⁻⁶mL/min, accuracy 0.2%) and three fluid storage transfer cylinders and a CO_2_ gas tank; the core holder system mainly consists of a core holder (core adapted length 5 ~ 8 cm) and a confining pressure pump (maximum confining pressure 70 MPa); the temperature control system can reach a maximum temperature of 150 °C; the NMR testing system is integrated with the displacement system, enabling online testing of core NMR porosity & permeability, T_1_/T_2_ spectra, and NMR imaging; the metering system includes an outlet pressure testing device and a fluid metering device; the data acquisition and analysis system mainly consists of a computer and data acquisition and post-processing software.

#### Experimental scheme and steps

The experimental research plan, with the goal of quantitatively analyzing the influence of injection pressure, temperature, pore structure, core permeability, and oil viscosity, was conducted using NMR testing. The experimental plan is shown in Table 2, and specific experimental steps are as follows:

**Table 2:** The plan of NMR displacement experiment.

Experiment groups	Core No.	Injection pressure (MPa)	Confining pressure(MPa)	Back pressure(MPa)	Temperature(℃)	Type of saturated oil
Group of injection pressure	10 − 1	15.0	18.0	14.5	60	#46
	10 − 2	20.0	23.0	19.5	60	#46
	10 − 3	25.0	28.0	24.5	60	#46
Group of temperature	9 − 1	20.0	23.0	19.5	40	#46
	9 − 2	20.0	23.0	19.5	60	#46
	9 − 3	20.0	23.0	19.5	80	#46
Group of pore structure	9 − 2	20.0	23.0	19.5	60	#46
	10 − 2	20.0	23.0	19.5	60	#46
Group of permeability	2 − 1	20.0	23.0	19.5	60	#46
	9 − 2	20.0	23.0	19.5	60	#46
	4 − 1	20.0	23.0	19.5	60	#46
Group of oil viscosity	7 − 1	20.0	23.0	19.5	60	#46
	8 − 1	20.0	23.0	19.5	60	#100

(1) Place the required core samples in a drying oven and dry at 105 °C for 24 h to completely remove moisture from the cores, and measure their dry weight.

(2) Place the samples in a vacuum pressure saturation device, evacuate, and saturate with the white oil type specified in the scheme with a saturation pressure of 20 MPa for 24 h. After that, remove the core and measure its wet weight.

(3) Connect the NMR testing system and the data acquisition and analysis system for self-check, place standard samples into the testing device for calibration, and use the established calibration curve to perform NMR porosity and initial T_2_ spectrum tests on the core samples.

(4) Wrap the sample with heat-shrink film, use a heat gun to secure it tightly to the displacement end plugs, place the wrapped core into the core holder system and connect the displacement pipelines.

(5) Add fluorocarbon oil to the confining pressure system, connect the confining pressure lines, and turn on the confining pressure pump and temperature control system. Adjust the confining pressure and temperature to the predetermined values in the scheme, simultaneously, open the back-pressure system and adjust the back pressure to the predetermined value.

(6) Open the gas injection device and the CO_2_ cylinder valve to fill the gas storage tank, use a booster pump to pressurize the gas, while adjusting the tank pressure and temperature to bring CO_2_ to a supercritical state. Displace each core for a cumulative time of 20 min after opening the valve to initiate the CO₂ flood, record the displacement pressure difference and crude oil recovery in real time. During this period, select 8 time points for core T_2_ relaxation spectrum testing and 4 time points for NMR imaging.

(7) After the oil displacement process is completed, turn off the gas injection device, gradually reduce the back pressure, unload the displacement line pressure to 0 MPa, and gradually reduce the confining pressure. After the confining pressure drops to a safe value, disconnect the confining pressure and displacement lines, remove the core sample from the holder, and measure the core mass after displacement.

(8) Repeat the NMR displacement experiments according to the experimental plan, and turn off all instruments after all sample tests are completed.

## Results and analysis

Based on the principles of NMR, we can determine the oil content in pores of different sizes within the core based on the peak changes in the T_2_ spectrum and the relaxation time, thereby inferring the fluid flow patterns inside the core. However, it should be noted that the current standard for classifying pore sizes based on relaxation time is not unified. In this study, pores corresponding to T_2_ < 1ms are defined as micropores, those with 1ms < T_2_ < 100ms are percolation pores (medium pores), and those with T_2_ > 100ms are migration pores (macropores)^35^. The following sections discuss the effects of injection pressure, temperature, core pore structure, permeability, and oil viscosity on the oil displacement efficiency under high-temperature and pressure injection conditions, categorized by experimental group.

### Group of injection pressure

**Fig. 4:**
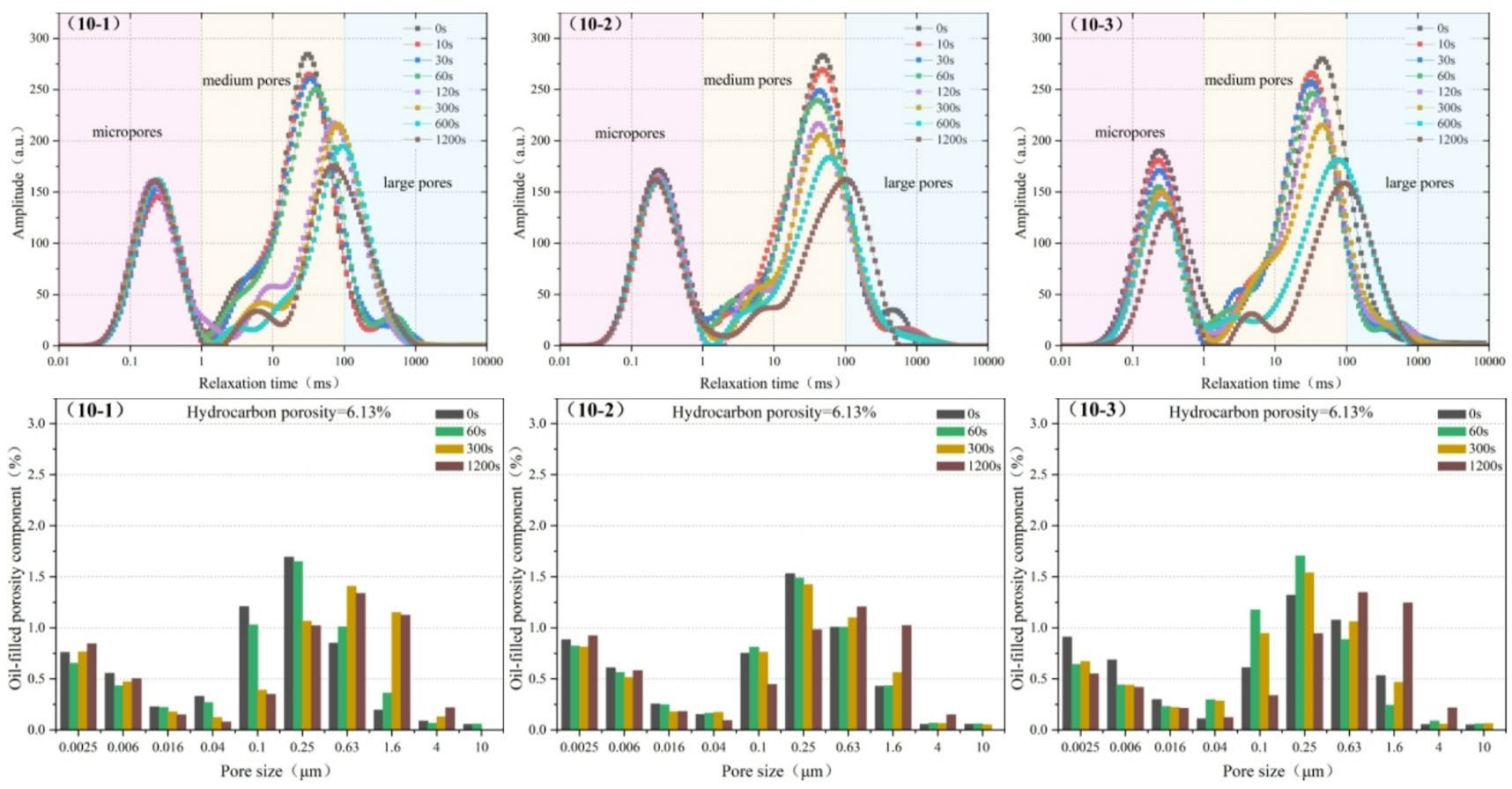
T_2_ relaxation spectrum and changes in oil porosity components of injection pressure group.

Core sample 10 was selected as the experimental core for the injection pressure group. The measured changes in the T_2_ relaxation spectrum and oil-filled porosity components are shown in Fig. 4, which indicates that the internal pores of core 10 are mainly medium pores according to the pore classification standard based on relaxation time. The oil signal decreased gradually with increasing displacement time, with the peak signals dropping from initial values of 280.5, 283.1, and 279.6 to 175.3, 162.3, and 158.7, respectively, during the three complete displacement processes. As the injection pressure increases, the oil content in the core decreases, and the entire peak shows a significant rightward shift, suggesting that the oil inside the core is gradually migrating towards larger pore spaces. Notably, when the displacement pressure reaches 25 MPa, the oil signal in the small pores also decreases slowly, indicating that the oil in pores of this size is gradually being extracted by the action of CO_2_.

The oil-filled porosity components show the changes in porosity occupied by different pore sizes and the changes in oil content. For core sample 10, pores with sizes in the range of 0.04 ~ 0.63 μm are the main storage space for oil, accounting for 60% of the total pore space. When the injection pressure is 15 MPa and 20 MPa, the oil content in the pore size range of 0.04 ~ 0.25 μm decreases, while the oil content in pores larger than 0.25 μm increases, indicates that CO_2_ promotes oil migration in the core. While the injection pressure reaches 25 MPa, the oil in the small pores decreases significantly at the beginning of the displacement, and the oil in the medium pores increases noticeably.

Weighing the core before and after the displacement experiment can determine the change in oil content in the core and further determine the oil recovery rate for each displacement. Table 3 shows that the mass of core 10 is nearly the same after three saturations, indicating that it has reached a fully saturated state each time. As the displacement pressure increases from 15 to 25 MPa, the final oil recovery rate shows a gradual increase, progressing from 25% to 36.2% and then 39.1%. Combined with Fig. 4, it can be inferred that the pressure increases from 15 MPa to 20 MPa mainly results in more oil being displaced from the large and medium pores, thereby increasing the recovery rate, while the pressure increases from 20 MPa to 25 MPa, the oil in the small pores is extracted under the action of CO_2_, however, because the total oil content in the small pores is lower, the increase in displacement efficiency is less pronounced.

**Table 3:** Changes in core quality and displacement efficiency (group of injection pressure).

Core No.	Dry weight (g)	Saturated weight (g)	Weight after displacement (g)	Displacement efficiency (%)
10 − 1	63.05	64.33	64.01	25.0
10 − 2	63.05	64.32	63.87	36.2
10 − 3	63.05	64.33	63.83	39.1

**Fig. 5:**
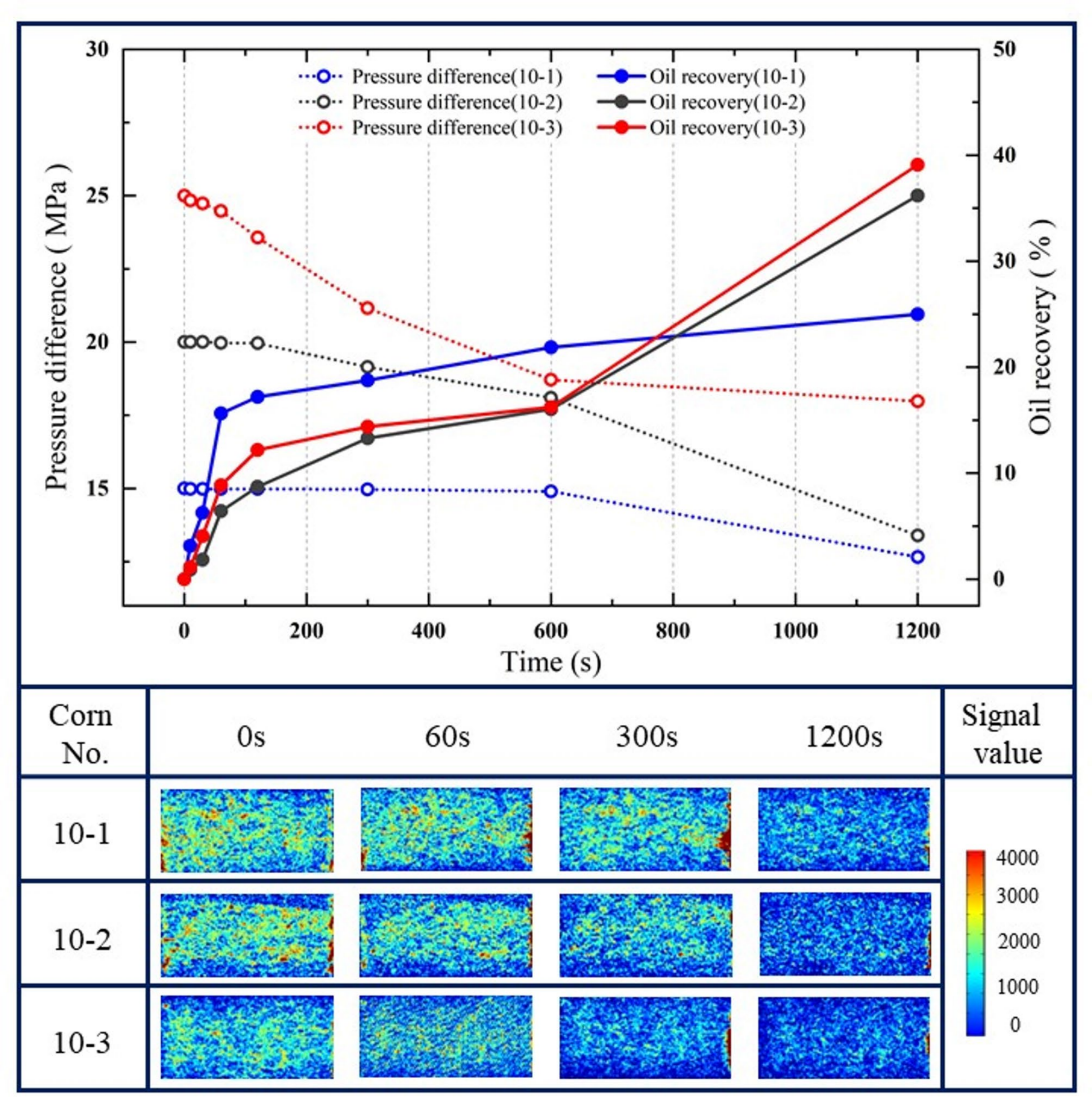
Displacement results and magnetic resonance imaging of injection pressure group.

**Fig. 6:**
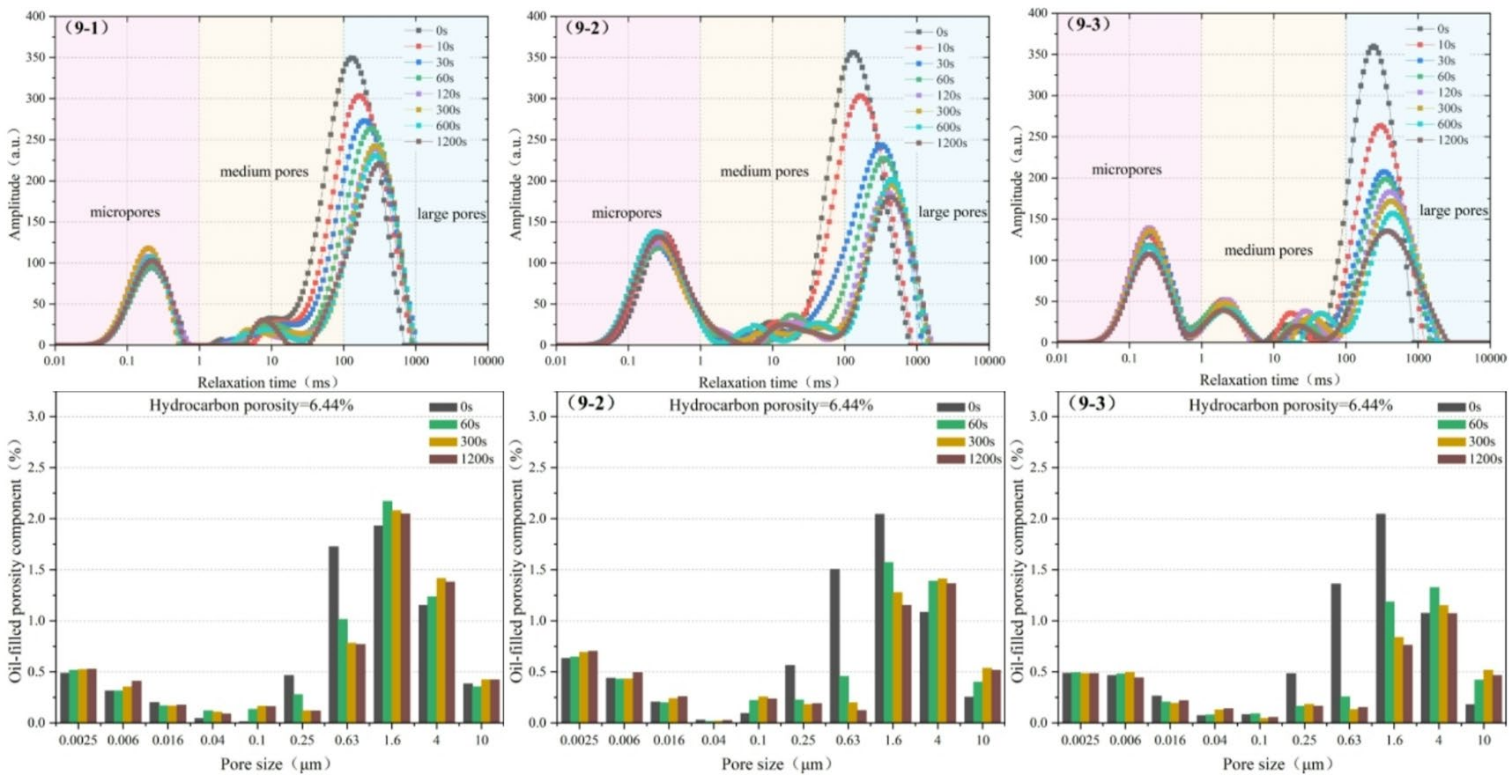
T_2_ relaxation spectrum and changes in oil porosity components of temperature group.

The displacement results and imaging diagrams shown in Figs. 5, 6 provide a more detailed description of the oil recovery efficiency and the internal oil distribution in the core. The change in displacement efficiency over time can be obtained from the final recovery rate combined with the T_2_ relaxation spectrum signal quantity, and the inlet-outlet pressure difference is calculated based on the inlet and outlet pressure values. The NMR imaging results during displacement are obtained after signal acquisition by the data acquisition system and processed by image analysis software, where red and yellow areas indicate higher oil signals, and blue and green areas indicate lower oil signals. When the injection pressure is 15 MPa, the inlet-outlet pressure difference hardly changes in the first 5 min of displacement. When the displacement time reaches 20 min, the outlet pressure increases, causing the pressure difference to decrease and reaching 2.34 MPa at the end of displacement, which indicates that the gas breakthrough time at the outlet under this injection pressure is between 5 ~ 20 min. As the injection pressure increases, the gas breakthrough time occurs earlier, with the outlet pressure under 20 MPa and 25 MPa beginning to rise after 2 min and just 10 s, respectively, and reaching 6.61 MPa and 7.02 MPa by the end of displacement. Imaging results indicate that as displacement proceeds, the oil signal decreases with the oil gathering toward the outlet end driven by CO_2_, and a higher displacement pressure yields a lower oil signal at identical time nodes. After 20 min of displacement, except for some oil accumulation at the outlet, there are still some bright areas inside core 10 − 1, while core 10 − 3 almost has no bright areas left, more intuitively reflecting that higher displacement pressure has a better displacement effect.

### Group of temperature

We selected core 9 for the temperature group, running multiple displacement experiments at 40 °C, 60 °C, and 80 °C under 20 MPa. The changes in the T_2_ relaxation spectrum and oil-filled porosity components of core 9 reveal two main peaks, with the first ranging from 0.1 to 1ms and the second, significantly higher, from 100 to 1000ms, indicating a pore structure dominated by large pores supplemented by some small pores. As displacement proceeds, it can be seen that the signal value in the large pores gradually decreases, while the signal value in the small pores changes little, indicating that only the oil in the large pores is effectively displaced. As displacement temperature increases, the signal amplitude of the T_2_ relaxation spectrum exhibits a more pronounced decrease over the same period, with the second main peak dropping from 349.4 to 220.9 at 40 °C compared to a much larger decrease from 360.0 to 135.5 at 80 °C, due to the significant reduction in oil viscosity which enhances mobility.

Based on the oil-filled porosity components, it is clear that pores larger than 0.25 μm in core 9 are the main storage space for oil, accounting for 76.8% of the total oil content. Analysis of the component changes shows that oil tends to migrate toward larger pores during displacement, with a significant decrease in oil content within the 0.25 ~ 1.6 μm pore size range as temperature increases. When the temperature is 40 °C, the oil content in pores ranging from 0.25 ~ 0.63 μm decreases by about half after displacement, while the oil content in pores ranging from 0.63 ~ 1.6 μm does not change much. When the displacement temperature increases to 60 °C, the oil content in pores ranging from 0.25 ~ 0.63 μm approaches zero after displacement, and the oil content in pores ranging from 0.63 ~ 1.6 μm decreases by about 44%. When displacement is carried out at 80 °C, the oil content in this pore size range decreases by about 62%. However, for pores with sizes less than 0.1 μm, temperature changes do not significantly affect their oil content.

The changes in core quality and displacement efficiency before and after displacement for the temperature group are shown in Table 4. With the temperature rising from 40 °C to 60 °C and then to 80 °C under 20 MPa, the oil recovery rate increased from a baseline of 62.6% by 6.9% and 11.6%, respectively, thereby confirming that the oil displacement efficiency improves with increasing temperature under the same injection pressure.

**Table 4:** Changes in core quality and displacement efficiency (group of temperature).

Core No.	Dry weight (g)	Saturated weight (g)	Weight after displacement (g)	Displacement efficiency (%)
9 − 1	58.52	59.83	59.01	62.6
9 − 2	58.52	59.80	58.91	69.5
9 − 3	58.52	59.84	58.86	74.2

**Fig. 7:**
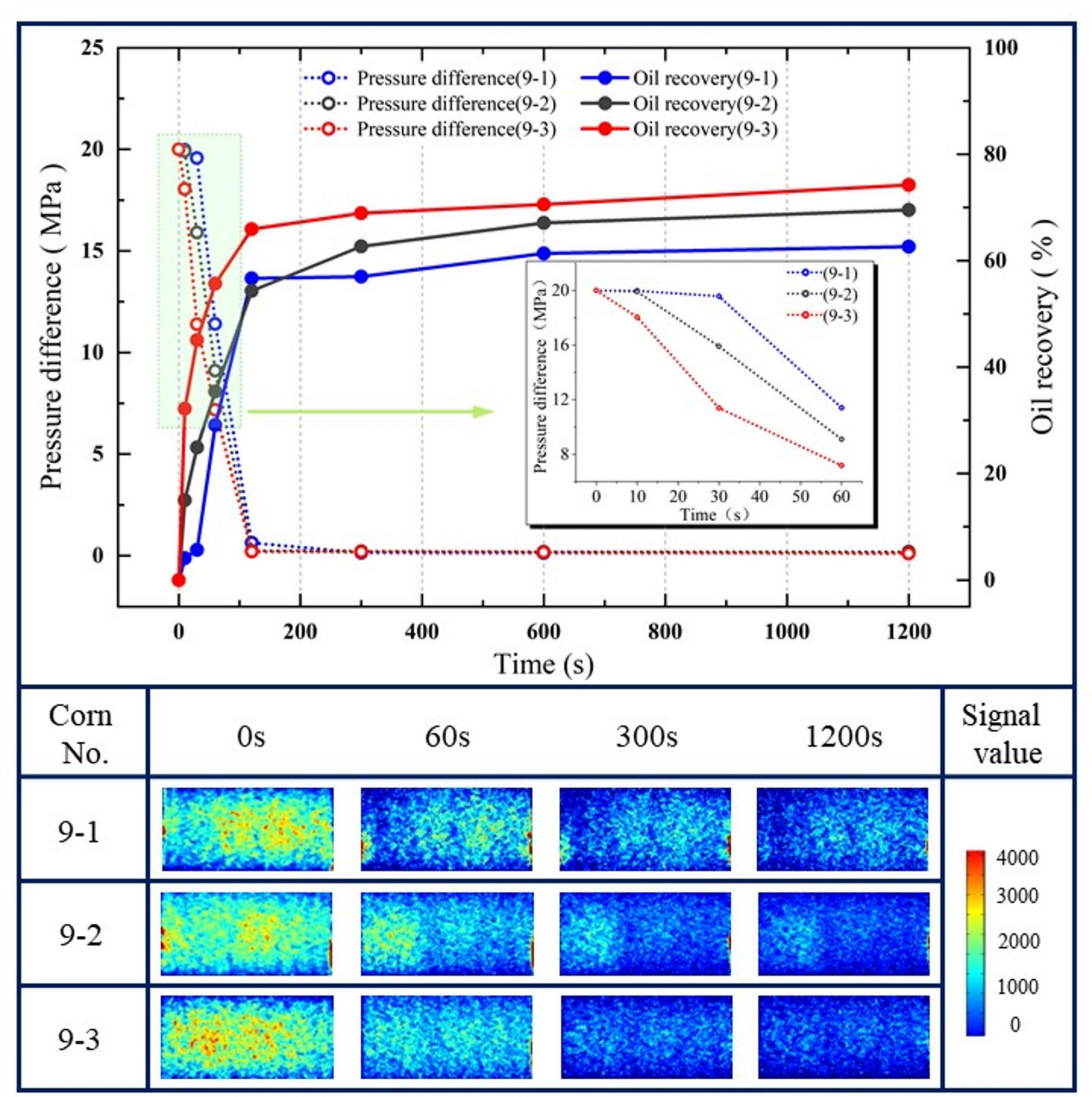
Displacement results and magnetic resonance imaging of temperature group.

**Fig. 8:**
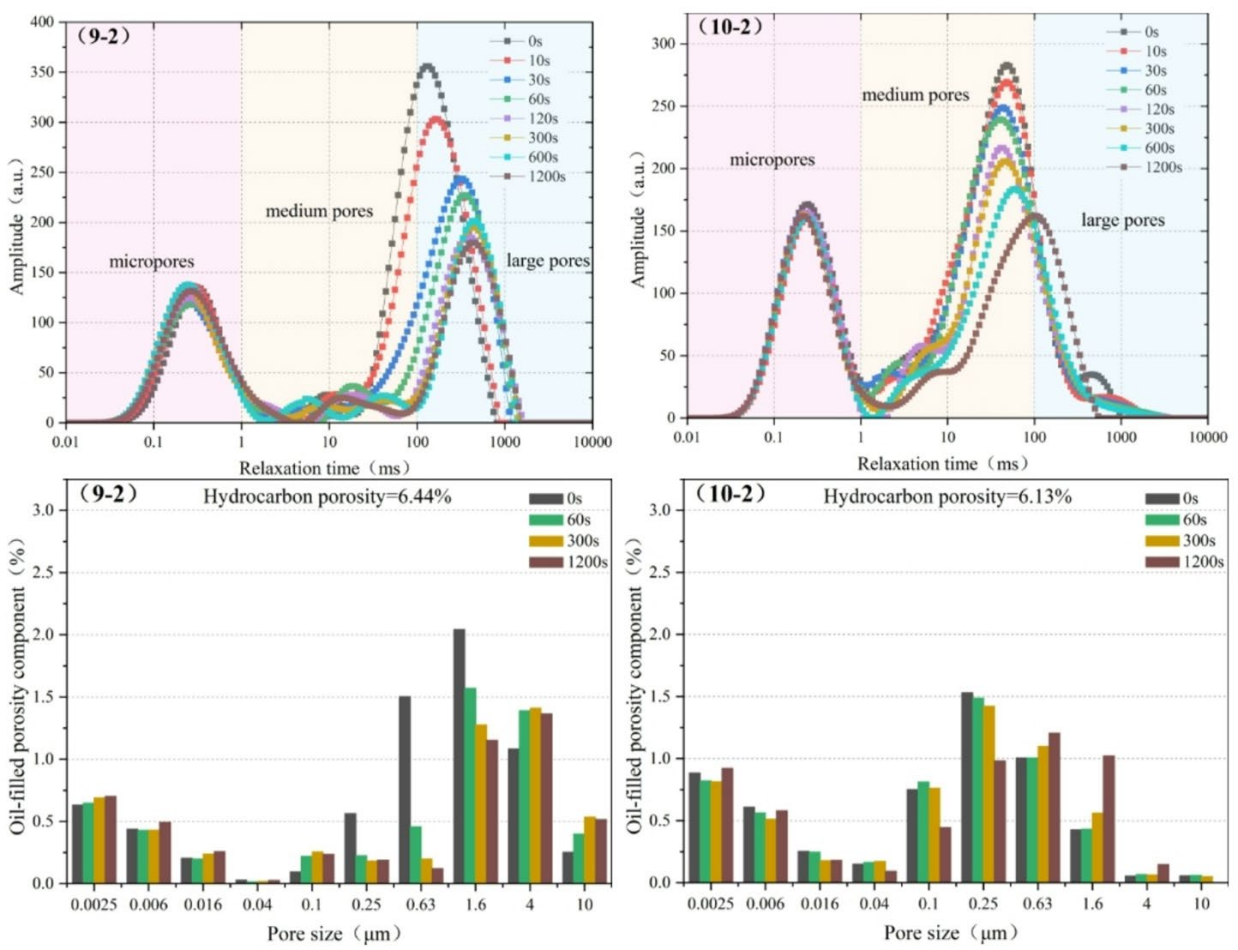
T_2_ relaxation spectrum and changes in oil porosity components of pore structure group.

According to the displacement results and imaging diagrams shown in Figs. 7, 8, the pressure drop during the three displacement processes of core 9 is rapid but still different. Based on the enlarged view of the ports pressure difference—where gas breakthrough occurred after 30 s for core 9 − 1, after 10 s for core 9 − 2, and immediately at the start of displacement for core 9 − 3—it is demonstrated that increasing the temperature accelerates CO_2_ gas breakthrough, thereby establishing displacement pathways within the core earlier. According to the change curve of the oil recovery rate, during the three displacement processes of core 9, the recovery rate shows a trend of rapid increase followed by a slow increase. The NMR images show a significant decrease in bright areas after one minute of displacement, indicating that most of the oil in the core has been produced, and also reveal that the bright areas in core 9 − 3 are significantly less than those in core 9 − 1. Based on the above analysis, under the same experimental conditions, the higher the temperature, the faster the outlet pressure rises, the higher the CO_2_ displacement efficiency, and the more oil is displaced from the core in the same time.

### Group of pore structure

To analyze the influence of core pore structure on CO_2_ flooding, we selected core 9 (oil-filled porosity 6.44%) and core 10 (oil-filled porosity 6.13%), which have similar porosities but different pore structures, as controls, under the same experimental conditions (injection pressure 20 MPa, temperature 60 °C). Core 9, with its second main peak in the 100 ~ 1000ms range, is dominated by large pores and contains most of its oil in pores larger than 0.25 μm, while Core 10, with its second peak in the 10 ~ 100ms range, is dominated by medium pores, with oil primarily located in the 0.04 ~ 0.25 μm range. Therefore, analyzing the T_2_ relaxation spectra and changes in oil-filled porosity of these two cores during CO_2_ flooding can reveal the oil distribution and migration patterns in cores with different pore structures.

The results demonstrate that the second peak signal of core 9 − 2 decreases faster early on, with a total drop from 355.9 to 179.8, in contrast to the gradual decrease from 283.1 to 162.3 for core 10 − 2, demonstrating that the greater reduction for core 9 − 2 signifies more oil was displaced. Combined with the changes in oil-filled porosity components, it can be seen that in the early stage of displacement, the oil content in pores ranging from 0.25 ~ 1.6 μm in core 9 − 2 decreases significantly, while the oil content in pores ranging from 0.04 ~ 0.25 μm in core 10 − 2 decreases significantly only after a period of displacement, indicating that a good pore structure promotes oil production.

The mass changes and displacement efficiency before and after displacement for cores 9 − 2 and 10 − 2 are shown in Table 5. Although the saturated oil amounts of cores 9 − 2 and 10 − 2 were nearly identical at 1.28 g and 1.27 g, respectively, the displaced oil amount and displacement efficiency differed significantly—0.89 g (69.5%) for core 9 − 2 compared to only 0.45 g (36.2%) for core 10 − 2—demonstrating that the core pore structure greatly impacts CO_2_ flooding efficiency and that a favorable pore structure is key to determining the oil recovery rate.

**Table 5:** Changes in core quality and displacement efficiency (group of pore structure).

Core No.	Dry weight (g)	Saturated weight (g)	Weight after displacement (g)	Displacement efficiency (%)
9 − 2	58.52	59.80	58.91	69.5
10 − 2	63.05	64.32	63.87	36.2

**Fig. 9:**
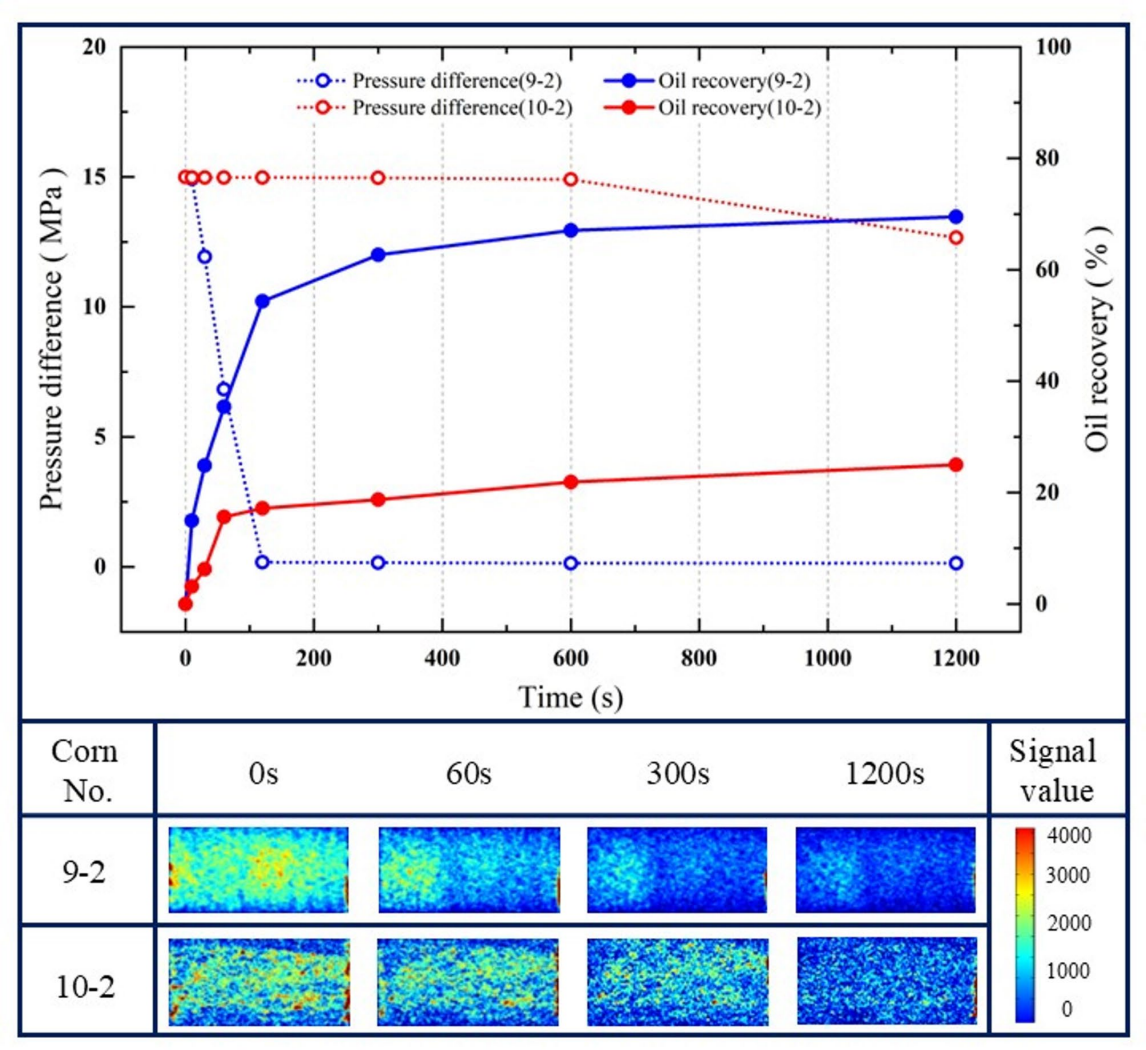
Displacement results and magnetic resonance imaging of pore structure group.

The displacement results and NMR imaging (Fig. 9) clearly show the differences between the two during displacement. During the displacement of core 9− 2, the oil recovery rate increased rapidly as the outlet pressure increased, reaching 54.3% after 2 min when the port pressures equalized—indicating complete core connectivity—after which the growth rate slowed, whereas core 10 − 2 exhibited a final recovery rate of only 36.2%, significantly lower than that of core 9 − 2. The NMR imaging more clearly shows that after 1 min of displacement of core 9 − 2, the bright areas in the imaging diagram have significantly reduced, indicating that a considerable amount of oil has been displaced from the core, while the imaging diagram of core 10 − 2 still has large bright areas even after 5 min of displacement, indicating that there is still a lot of oil inside the core that has not been effectively displaced. Based on the above analysis, it can be seen that a higher proportion of large-sized pores not only makes it easier to establish displacement pathways and achieve higher recovery under the same conditions, but its degree of influence also far exceeds that of injection pressure and temperature.

### Group of permeability

The test results show that cores 2 − 1, 9 − 2, and 4 − 1 have similar pore structures, with gas permeabilities of 3.54md, 0.27md, and 0.03md, respectively, differing by an order of magnitude from each other, so it is reasonable to use these three cores as the permeability group core displacement samples in the experimental plan.

**Fig. 10:**
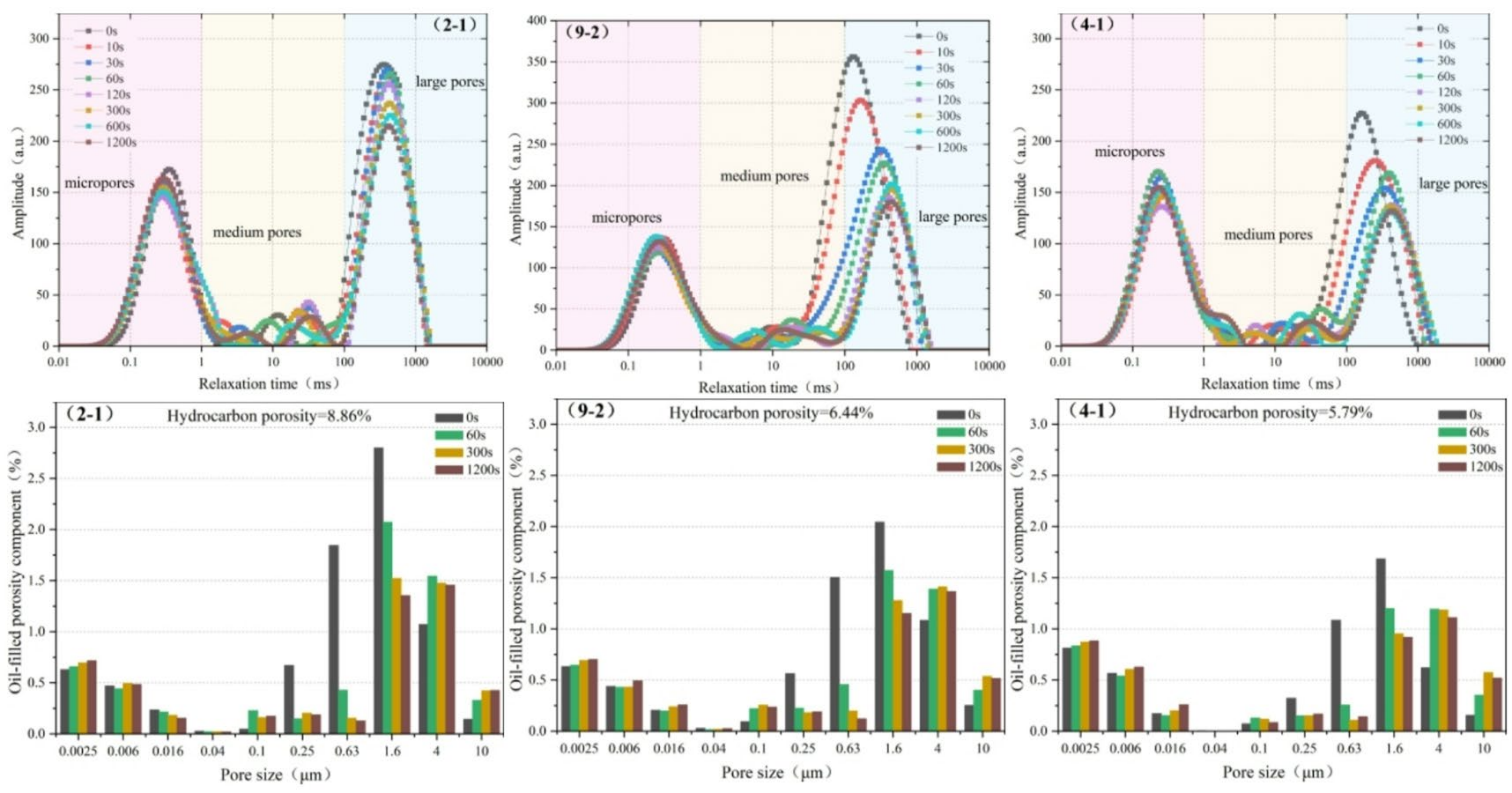
T_2_ relaxation spectrum and changes in oil porosity components of permeability group.

Figure 10 reveals similar peak change trends in the T_2_ relaxation spectra and oil-filled porosity components across the three cores, characterized by a greater signal decrease early in displacement and a lesser one later, owing to their internally large pore dominated structures. Throughout the displacement process, the main peak signals decreased from 373.4 to 167.6 for core 2 − 1, from 355.9 to 179.8 for core 9 − 2, and from 227.4 to 131.8 for core 4 − 1, with the greater signal decrease correlating to higher core permeability. This is primarily because, for cores with similar pore structure, higher permeability not only indicates larger pore space, more saturated oil, and a stronger initial NMR peak signal, but also better pore connectivity, which is more conducive to oil recovery and thus results in a greater signal decrease. Analysis of the oil-filled porosity components shows that while the oil content in the 0.1 ~ 1.6 μm pores decreases significantly after one minute of displacement in all three cores, the oil in smaller pores remains largely unchanged regardless of permeability. For tight cores with few pores and poor connectivity, while methods like fracturing can enhance permeability and improve connectivity, they cannot alter the fundamental pore structure, making it necessary to subsequently increase displacement pressure to effectively recover oil from small pores.

Table 6 shows that cores 2 − 1, 9 − 2, and 4 − 1 all have displacement efficiencies exceeding 60%, with their respective saturated oil amounts being 1.54 g, 1.28 g, and 1.01 g; displaced oil amounts 1.11 g, 0.89 g, and 0.66 g; and efficiencies 72.1%, 69.5%, and 65.3%. It can be seen that while higher core permeability leads to higher displacement efficiency—increasing by only about 2 ~ 4% per order of magnitude increase, as observed in the experimental cores—this improvement is smaller in effect compared to the influence of pore structure, although it confirms that increasing permeability aids oil production.

**Table 6:** Changes in core quality and displacement efficiency (group of permeability).

Core No.	Dry weight (g)	Saturated weight (g)	Weight after displacement (g)	Displacement efficiency (%)
2 − 1	59.31	60.85	59.74	72.1
9 − 2	58.52	59.80	58.91	69.5
4 − 1	61.50	62.51	61.85	65.3

**Fig. 11:**
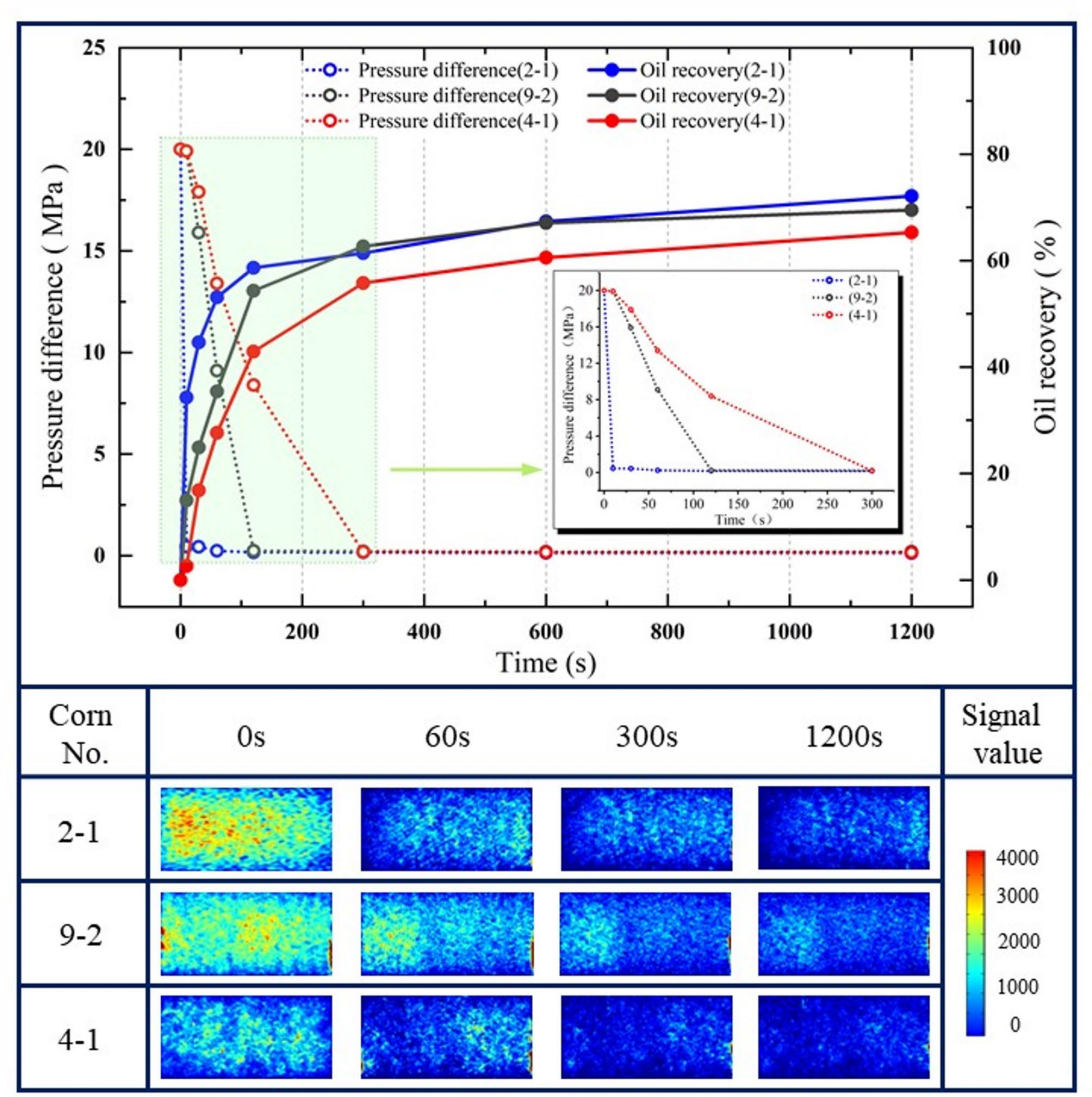
Displacement results and magnetic resonance imaging of permeability group.

**Fig. 12:**
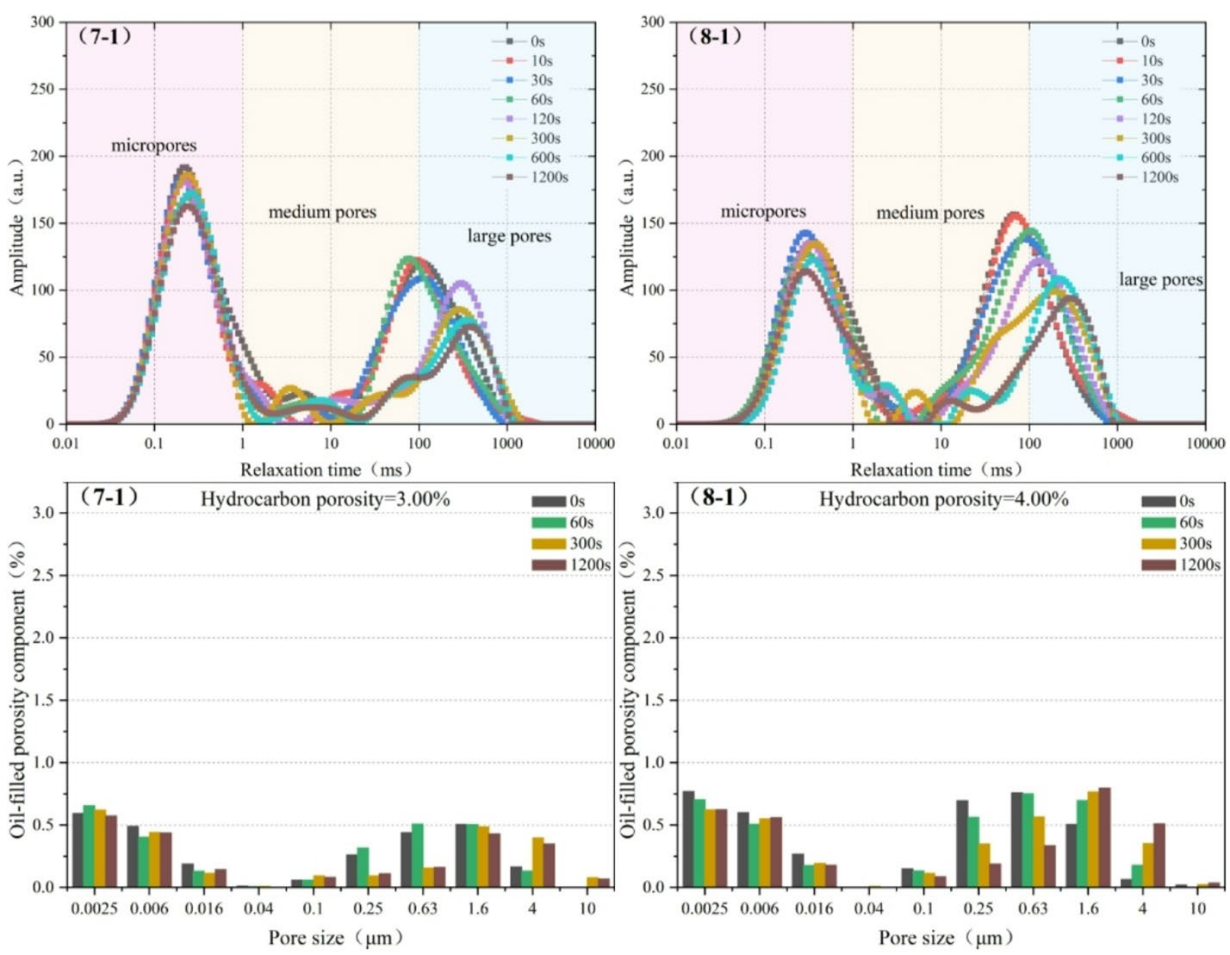
T_2_ relaxation spectrum and changes in oil porosity components of oil viscosity group.

The core displacement results and NMR imaging of the permeability group (Figs. 11, 12) show that the inlet-outlet pressure differences of cores 2 − 1, 9 − 2, and 4 − 1 all decrease by different amounts in the early stage, with core 2 − 1 dropping to near 0 after 10 s; core 9 − 2, the next fastest, after 120 s; and core 4 − 1, the slowest, only after 300s. This is because higher core permeability indicates better pore connectivity in the core, which is more conducive to fluid flow and diffusion between pores, causing CO_2_ to quickly pass through the core and prompting a sharp increase in the outlet pressure value. Although the oil recovery rate curves for all three cores show a rapid-then-slow increasing trend with similar final efficiencies, the difference in core permeability primarily affects the growth rate during the increasing phase.

### Group of oil viscosity

We selected cores 7 − 1 and 8 − 1 with similar pore structure and permeability for the oil viscosity group, with core 7 − 1 saturated with #46 white oil under 20 MPa for 24 h and core 8 − 1 saturated with #100 white oil under 20 MPa for 24 h.

The changes in the T_2_ relaxation spectrum indicate similar peak signal evolution for cores 7 − 1 and 8 − 1 during displacement, with the first peak remains largely stable while the second exhibits a right-shifting and gradual decrease, revealing that only oil in medium and large pores is effectively displaced. Due to pore space constraints, the overall oil content in cores 7− 1 and 8 − 1 is relatively low. With the content in pores smaller than 0.63 μm decreasing and that in pores larger than 1.6 μm increasing as displacement proceeds, demonstrating that oil gradually migrates toward and is produced from larger pores under CO_2_ displacement. Based on the above analysis, it is believed that the difference in oil viscosity does not significantly affect the migration and distribution patterns of oil in the pores.

The data in Table 7 show that the saturated oil amount, displaced oil amount, and displacement efficiency are 0.47 g, 0.28 g, and 59.6% for core 7 − 1 and 0.57 g, 0.27 g, and 47.4% for core 8 − 1, respectively. Reducing oil viscosity has a certain promoting effect on CO_2_ flooding, as observed by the relatively higher efficiency in core 7 − 1 (saturated with lower-viscosity #46 white oil) and the about 12% decrease for core 8 − 1 with doubled oil viscosity, even though the pore structures of the two cores are not completely identical.

**Table 7:** Changes in core quality and displacement efficiency (oil viscosity group).

Core No.	Dry weight (g)	Saturated weight (g)	Weight after displacement (g)	Displacement efficiency (%)
7 − 1	60.58	61.05	60.77	59.6
8 − 1	60.32	60.89	60.62	47.4

**Fig. 13:**
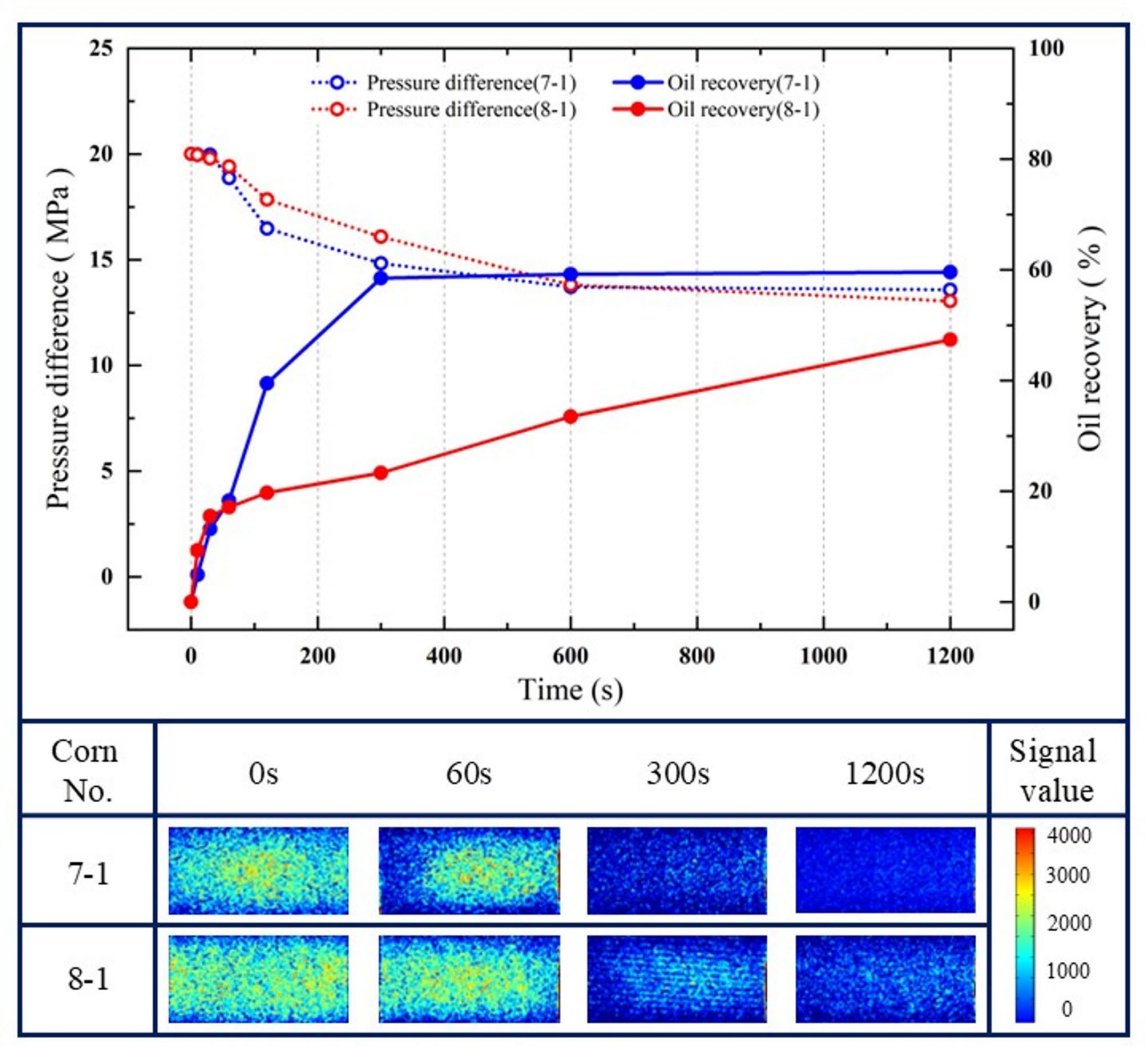
Displacement results and magnetic resonance imaging of oil viscosity group.

As shown in Fig. 13, the differential pressure across the cores of core 7 − 1 and core 8 − 1 decreased rapidly after gas breakthrough and then stabilized. At 20 min, the inlet-outlet differential pressure of core 7 − 1 was 13.58 MPa, and that of core 8 − 1 was 13.05 MPa, indicating that the outlet pressure values were very close. However, their oil recovery rates change quite differently: core 7 − 1 rapidly increased to 58.19% within the first 5 min, very close to its final efficiency of 59.6%, while core 8 − 1 had a rapid production stage lasting only 0.5 min, after which it maintained a relatively fixed increase until the end of displacement, reaching a final recovery rate of 47.4%. It is indicated that under similar core pore structures, lower oil viscosity facilitates rapid oil production, thereby demonstrating that reducing oil viscosity does help improve the recovery factor to a certain extent. The NMR imaging results show that the oil gradually migrates toward the outlet during displacement, with the bright areas in both cores significantly shrinking after 5 min. Based on the above analysis, higher oil viscosity adversely impacts CO_2_ flooding efficiency in that it makes production more difficult, specifically manifested as a shorter rapid production stage and a relatively lower oil recovery rate over the same period.

### Analysis of influencing factors

**Fig. 14:**
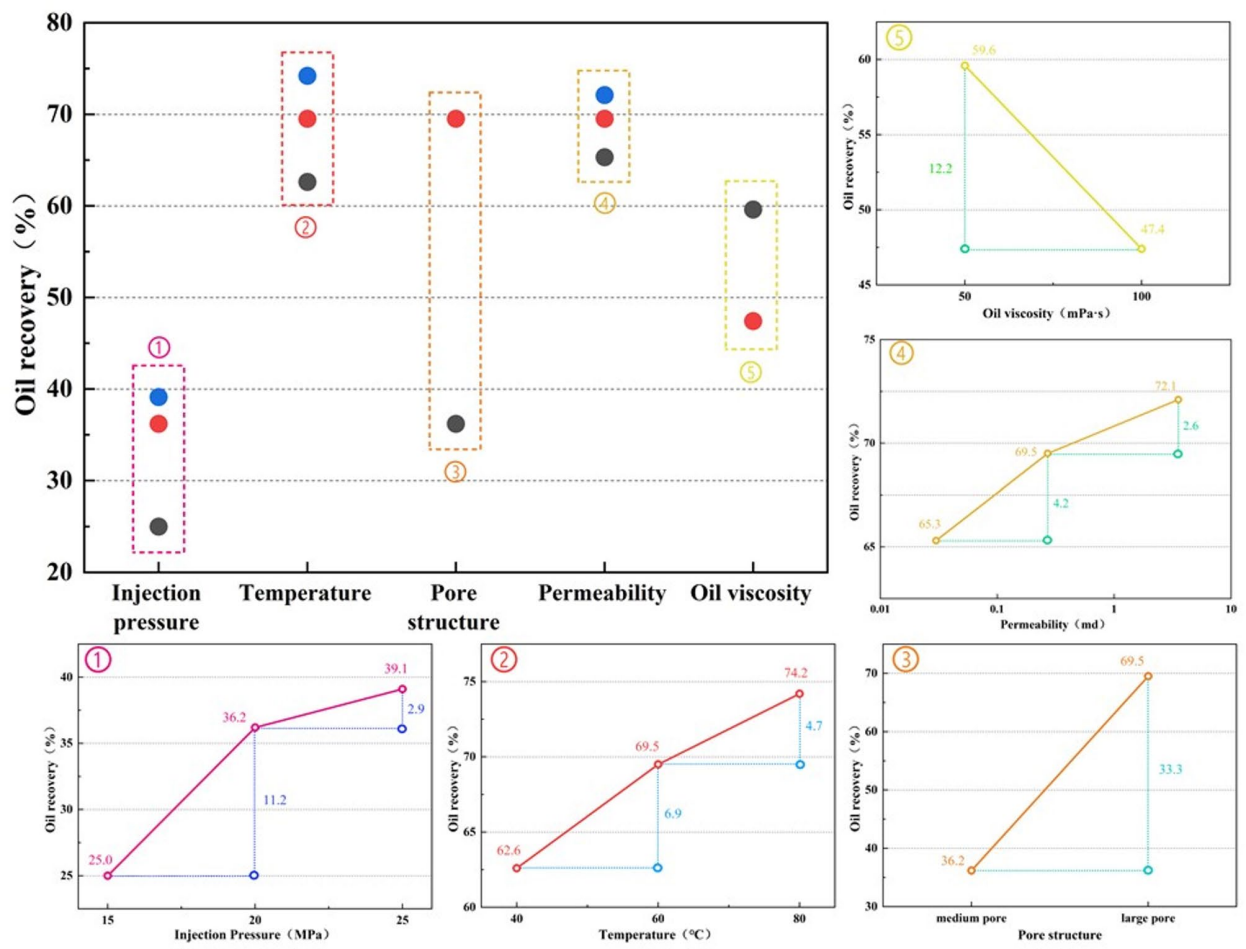
Evaluation of the ultimate oil recovery factor under varying experimental conditions.

To further study the influence of various factors on oil recovery efficiency, the final oil recovery rates under different experimental conditions are summarized and analyzed (Fig. 14).

The results show that rock pore structure is the key factor affecting the oil recovery rate, as it directly determines the oil storage space and type, with the recovery rate nearly doubling as the dominant pore structure transitions from medium to large pores. A good pore structure often means a larger storage space and a larger proportion of movable oil; cores with poor pore structure often have mainly tiny pores internally, with small pore space and most of the oil existing in the core as irreducible oil.

The relationship between injection pressure and oil recovery is nonlinear: at lower pressures, CO_2_ can displace oil from medium and large pores, whereas at pressures reaching 25 MPa, CO_2_ can effectively extract oil from small pores. However, since this part of the oil itself accounts for a relatively low proportion, continuously increasing the pressure at this point cannot significantly improve the recovery rate.

The relationship between temperature and oil recovery is nearly linear, as the temperature increase significantly reduces crude oil viscosity and enhances its fluidity, thus improving the recovery rate, with each 20 °C rise resulting in an approximately 5 ~ 7% increase in recovery.

The difference in oil viscosity directly affects the recovery rate, as demonstrated by the 12.2% difference in recovery efficiency between the 50mPa·s and 100mPa·s white oils used in the experiment, which approximately represent medium and high viscosity crude oils respectively.

The influence of permeability on recovery rate in this experiment differs notably from previous studies, as evidenced by only a 6.8% difference in recovery despite a two order of magnitude variation in core permeability, which may be attributed to insufficient control over the influence of pore structure in earlier research. Generally, cores with high permeability have better pore structures, whereas the cores selected in this experiment have similar pore structures but greatly different permeabilities. Thus, our test results better avoid the influence of pore structure and can better reflect the influence of core permeability on the recovery rate.

## Conclusion

We studied the effects of injection pressure, temperature, pore structure, permeability, oil viscosity, and other factors on CO_2_ flooding through CO_2_ flooding experiments, visualized the flooding process, revealed the distribution and migration patterns of oil in pores of various scales under different experimental conditions, as well as the changes in core inlet-outlet pressure difference and oil recovery rate by NMR testing, and conducted a comprehensive analysis and summary:

(1) CO_2_ is an effective displacement medium. Throughout the displacement process, the peak signal of the core T_2_ relaxation spectrum gradually decreases and shifts to the right, indicating that under the displacement action of CO_2_, the oil in the core migrates towards larger pores and is gradually displaced from the core.

(2) The experimental results show that injection pressure is one of the key factors affecting CO_2_ flooding, as the higher the injection pressure, the higher the displacement efficiency of CO_2_. When the pressure is above 25 MPa, CO_2_ can effectively extract oil from the small pores of the core, resulting in a recovery rate of 39.1%, which is of great significance to improve crude oil production rate in tight reservoirs.

(3) The influence of temperature on CO_2_ oil recovery mainly lies in the fact that increasing temperature can effectively reduce oil viscosity, accelerate fluid flow in the core, and thus improve oil recovery efficiency. The changes in the core T_2_ relaxation spectrum, oil-filled porosity components, and NMR imaging show that under a certain displacement pressure, the higher the temperature, the more oil is displaced from the core in the same displacement time. However, it has little impact on the oil recovery effect of core small pores, which indicates that the recovery improvement of thermal injection development is limited.

(4) Pore structure is the most important factor determining CO_2_ flooding efficiency. The experimental results show that displacement pathways are more easily established in cores with a higher proportion of large pores. For cores with the same pore structure, higher permeability generally means good connectivity of pores, which is manifested in displacement experiments as a faster drop in outlet pressure and a higher oil recovery rate in the same time, but its impact on the recovery rate is much smaller than that of pore structure. For every order of magnitude difference in permeability, the recovery rate only increases by about 3 ~ 4%, which is significantly different from previous studies. Certainly, additional experimental batches are also needed to verify the accuracy of this conclusion.

(5) Generally, higher oil viscosity means slower flow in the core, which is not conducive to oil production. From the changes in T_2_ relaxation spectrum and oil-filled porosity components, it can be seen that the distribution and migration patterns of oils with different viscosities in the core are similar. However, in terms of displacement efficiency, oil with lower viscosity has better recovery performance, as the saturated oil viscosity of cores 7 and 8 differs by a factor of two, and the recovery factor differs by about 12%.

## Data Availability

All data supporting the findings of this study are available within the paper.
